# Lung Volume Changes in Stable Preterm Infants Weaned From Nasal CPAP to High Flow: A Prospective Cohort Study

**DOI:** 10.1016/j.chpulm.2024.100094

**Published:** 2024-08-14

**Authors:** Vanessa L. Büchler, Vincent D. Gaertner, Janine Thomann, Dirk Bassler, Christoph M. Rüegger

**Affiliations:** a Newborn Research, Department of Neonatology, University Hospital and University of Zürich, Zürich, Switzerland; b Division of Neonatology, Dr von Hauner Children’s Hospital, Ludwig-Maximilians-University Munich, Munich, Germany

**Keywords:** electrical impedance tomography, heated, humidified, high-flow nasal cannula, nasal CPAP, nasal high-flow therapy, preterm infant

## Abstract

**Background:**

Weaning preterm infants off nasal CPAP (nCPAP) using nasal high-flow therapy has gained popularity. The effects of such a weaning strategy on lung volumes are unclear.

**Research Question:**

How does the transition from nCPAP to high flow and varying flow levels affect lung volumes in stable preterm infants?

**Study Design and Methods:**

This was a prospective cohort study in infants 30 to 35 weeks’ postmenstrual age. After a baseline period on nCPAP 5 cm H_2_O, infants were switched to high flow 8 L/min for 30 minutes. The flow level was reduced by 2 L/min every 30 minutes to a minimum of 2 L/min and subsequently increased to the initial level of 8 L/min, followed by another nCPAP period. Using electrical impedance tomography, end-expiratory lung impedance as a proxy for end-expiratory lung volume (EELV) and cardiorespiratory parameters were recorded at each flow level and compared with baseline.

**Results:**

Overall, 8,438 breaths from 19 infants were analyzed. EELV changed significantly during the study (*P* = .002), which was mainly attributable to a loss of EELV when high flow was reduced to 6 and 4 L/min and re-escalated to 4, 6, and 8 L/min. Apart from a reduction in minute ventilation (*P* = .004), no other significant changes were found in electrical impedance tomography ventilation parameters. Alterations in lung volume were accompanied by an increase in heart rate (*P* = .02) and a decrease in peripheral oxygen saturation/Fio_2_ ratio (*P* < .001).

**Interpretation:**

The results of this study indicate that the transition from nCPAP to high flow is likely to result in a reduced EELV, accompanied by physiological responses in heart rate and oxygenation. Despite a stepwise escalation to preweaning flow levels, we found that only partial recovery of lung volume losses was achievable with high flow.

**Clinical Trial Registration:**

ClinicalTrials.gov; No.: NCT05237622; URL: www.clinicaltrials.gov

**Take-home Points**

**Study Question:** What are the alterations in lung volume observed in preterm neonates during the transition from nasal CPAP to high-flow therapy at varying flow rates?

**Results:** The transition from nasal CPAP to high-flow therapy, followed by subsequent high-flow weaning, was associated with gradual losses in lung volume. Although a stepwise escalation to preweaning flow levels allowed for partial recovery, complete restoration of lung volume was not achieved with high flow.

**Interpretation:** Our findings underscore the limitations of high-flow therapy in restoring lost lung volume. To preserve alveolar stability and address collapsed lung regions, pressures beyond those provided by high flow may be warranted.

Treatment with heated, humidified, high-flow nasal cannulae (nasal high flow) has gained popularity as an alternative strategy to nasal CPAP (nCPAP) for respiratory support of preterm infants.^1^, ^2^, ^3^ High flow promotes infant comfort, facilitates feeding and oral care, allows for gradual weaning, and reduces the risk of complications associated with nCPAP (eg, nasal trauma, air leaks).^4^ Despite the perceived benefits, evidence suggests that high-flow therapy may be less effective than nCPAP for primary and postextubation support of preterm infants.^5^, ^6^, ^7^ In addition, high-flow use has been associated with a prolonged duration of oxygen supplementation, despite facilitating a faster weaning process from nCPAP.^2^^,^^8^

Current nCPAP weaning protocols for the transition to high flow focus on infants achieving respiratory stability with low oxygen requirements, typically at distending pressures of 5 to 6 cm H_2_O.^9^ In contrast to nCPAP, high flow provides highly variable distending pressures that have not been reliably predictive of end-expiratory lung volume (EELV), potentially hindering the success of nCPAP weaning.^10^^,^^11^ Although there is a strong association between flow rate and the pressure generated in the pharynx during high-flow therapy in preterm infants, the effects of different flow levels on EELV remain poorly understood.^11^

The primary aim of this study was to describe the overall changes in lung volume during and after the transition to high-flow therapy and at varying flow levels. We assessed global end-expiratory lung impedance (EELZ) as a marker of EELV using electrical impedance tomography (EIT).^12^ EIT is a noninvasive, radiation-free imaging method used for bedside assessment of regional lung function. Electrodes placed around the chest circumference allow for the measurement of changes in bioelectrical impedance, enabling investigation of regional lung aeration and ventilation distribution on a breath-by-breath basis.^13^ We hypothesized that changes in flow levels would be positively correlated with changes in EELV.

## Study Design and Methods

This was a prospective cohort study, performed at the neonatal intermediate care unit of the University Hospital Zurich (Switzerland) between February and December 2022. This study was approved by the local ethics committee (BASEC-Nr. 2021-02454) and prospectively registered at ClinicalTrials.gov (NCT05237622). Written informed consent was provided by all parents prior to study start.

### Population and Intervention

**e-Figure 1:**
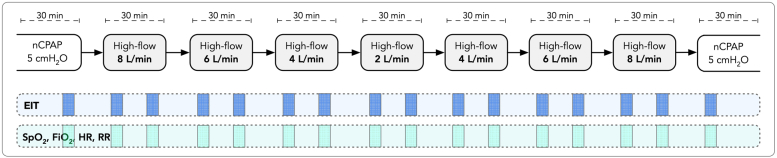


Preterm infants between 30 and 35 postmenstrual weeks who were (1) stable on nCPAP support with a positive end-expiratory pressure of 5 cm H_2_O and an Fio_2_ < 0.30 and (2) older than 72 hours were eligible for inclusion in the study. Both modes of respiratory support were delivered through the same ventilators (Fabian; ACUTRONIC Medical Systems AG or EVE NEO; Fritz Stephan GmbH). Binasal prongs, nasal facemasks (both Heinen & Löwenstein), or nasal high-flow cannula (Optiflow System; Fisher and Paykel) were used during nCPAP and high-flow support, respectively. During the last nursing care, infants were positioned prone in accordance with our internal guideline for preterm infants requiring respiratory support.^14^ Feeding intervals were scheduled to ensure that no feeds were administered during the intervention phase of the study. After the baseline period on nCPAP, infants were switched to high-flow support starting with a level of 8 L/min for 30 minutes. The initial flow rate of 8 L/min has been shown to generate a distending pressure similar to the previous nCPAP.^15^ The support level was gradually reduced by 2 L/min every 30 minutes to a minimum of 2 L/min. Subsequently, it was incrementally increased by 2 L/min every 30 minutes until it reached the initial level of 8 L/min. After an additional 30 minutes, the participants were switched back to nCPAP with a positive end-expiratory pressure of 5 cm H_2_O. No washout periods were provided between baseline and the subsequent high-flow levels. The entire study workflow is illustrated in e-Figure 1.

### Data Collection

The LuMon device (SenTec AG) was used to continuously record EIT data at a frame rate of 51 Hz.^16^ An electrode belt with 32 electrodes was placed around the chest at the height of the mamilla and left in place for the duration of the entire study.^17^ Heart rate and peripheral oxygen saturation (Spo_2_) were recorded continuously using a Masimo pulse oximeter set to 2-second averaging time and maximum sensitivity (Masimo Radical 7; Masimo Cooperation).

### Data Analysis

EIT data were extracted and analyzed using iBeX (version 1.1; SenTec AG) and MATLAB software (version 2019a; MathWorks). For the assessment of lung volume changes, predefined anatomic lung regions based on the vendor-provided human model chest atlas were projected into the EIT image and EIT signals outside these regions were excluded.^18^^,^^19^ At baseline, at the beginning and end of each high-flow level, and after the switch back to nCPAP, EIT signals from 30-second intervals of artifact-free tidal ventilation were extracted and normalized for body weight. For each 30-second interval, medians and interquartile ranges were used for further analysis.

### Outcomes

The primary outcome of this study was the change in global EELZ between nCPAP at baseline and high flow at different flow levels (ΔEELZ = EELZ_high flow_ – EELZ_nCPAP[baseline]_). Other EIT data (impedance change [surrogate for tidal volume, arbitrary units/kg/min], minute volume [arbitrary units/kg/min], center of ventilation [%] as a surrogate for ventilation distribution, silent spaces [%]), vital signs (heart rate, Spo_2_, Spo_2_/Fio_2_ ratio, respiratory rate) and Fio_2_, and adverse events during the study period (need for intubation, pneumothorax, treatment failure) were considered secondary outcomes. EIT-derived parameters were measured according to the 2016 Translational EIT Development Study Group Consensus,^13^ with definitions available in e-Table 1. Treatment failure was defined by the presence of one of the following criteria: (1) respiratory rate > 100 breaths/min for at least 30 minutes during the intervention, (2) increase in Fio_2_ by ≥ 0.25 from baseline to maintain oxygen saturation within predefined limits, and (3), > 2 apnea episodes requiring stimulation per 30-minute intervention.

### Statistical Analysis

Statistical analysis was performed using RStudio (Posit Software).^20^ Normally distributed data are presented as mean and SD, whereas nonparametric data are illustrated as median and interquartile range. Changes over time are described as differences from baseline assessment (nCPAP at start of the study) (ie, the median value at baseline was subtracted from the respective time point’s median value for each patient). Global differences in EELZ and other EIT and physiological parameters across different high-flow levels were assessed using the Friedman test. In case of statistically significant global tests, post hoc analyses were performed using paired Wilcoxon tests with Bonferroni-Holm correction for multiple comparisons. *P* < .05 was considered statistically significant. A convenience sample of 20 infants was chosen to ensure feasibility while providing sufficient information for clinically relevant findings.

## Results

**Figure 1:**
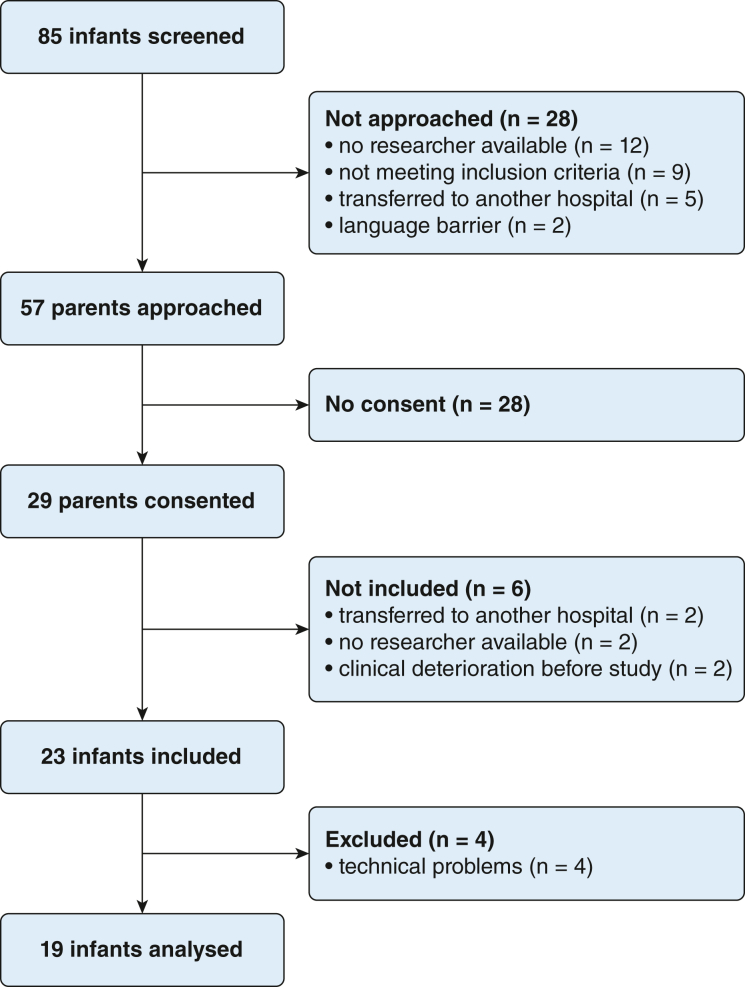
Flowchart of included infants.

Twenty-three infants were included in the study. Four infants were excluded due to technical difficulties (failure of EIT and data recording devices, insufficient electrode contact), leaving 19 infants for final analysis (Fig 1). Overall, 302 30-second intervals of artifact-free tidal ventilation including 8,438 breaths were analyzed. Demographic and clinical characteristics of included infants are presented in Table 1.

**Table 1:** Baseline Patient Characteristics (N = 19)

Patient Characteristics	Value
Prenatal	
Completed antenatal steroids	17 (90)
Chorioamnionitis	5 (26)
Perinatal	
Gestational age at birth, wk	28.1 (26.9-29.3)
Birth weight, g	1,110 (805-1,275)
Male	8 (42)
Apgar score at 5 min	8 (7-9)
Postnatal, before study	
Endotracheal ventilation	11 (58)
Duration of endotracheal ventilation, d	1 (0-2)
Exogenous surfactant	14 (74)
Duration of NIPPV, d	0 (0-2)
Duration of nCPAP, d	33 (23-39)
Supplemental oxygen, d	28 (5-46)
Postnatal steroids	2 (10)
Postnatal, at study	
Postnatal age, d	36 (27-47)
Postmenstrual age, wk	33.3 (32.0-34.1)
Weight, g	1,830 (1,550-2,100)
nCPAP pressure, cm H_2_O	5 (5-5)
Fio_2_	21 (21-24)
Caffeine dose, mg/kg/d	5 (5-5)

Values are depicted No. (%) or median and interquartile range. nCPAP = nasal CPAP; NIPPV = noninvasive positive pressure ventilation.

### Changes in EELZ

**Figure 2:**
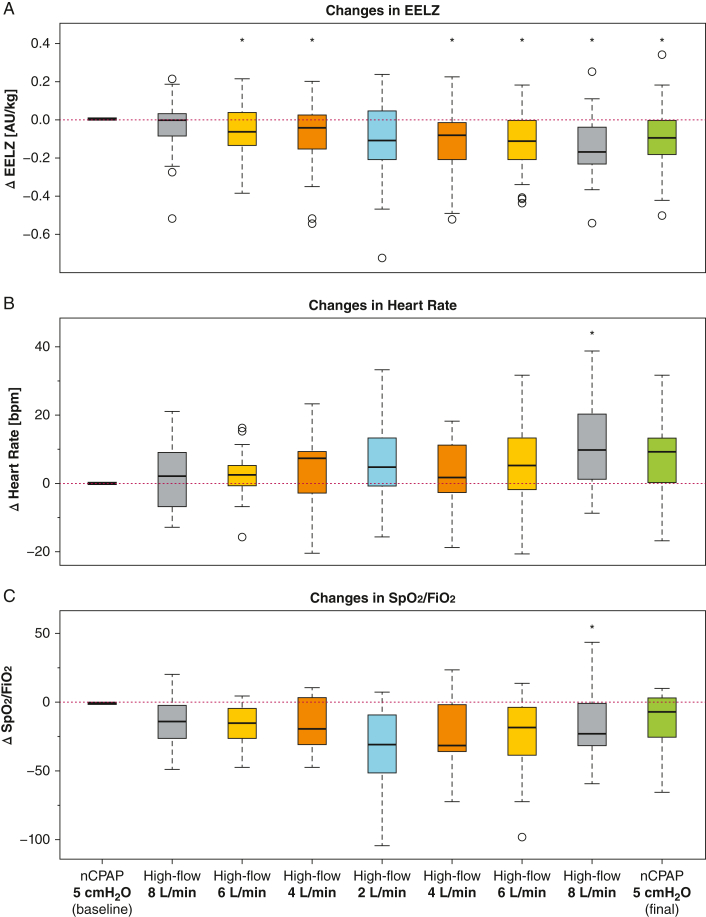
A-C, Changes in lung volume, heart rate, and Spo_2_/Fio_2_ ratio during the intervention period. A, Changes in end-expiratory lung impedance (EELZ) compared with nCPAP (baseline). Overall, EELZ changed significantly over time (Friedman test, *P* = .002). B, C, Changes in heart rate and Spo_2_/Fio_2_ ratio compared with nCPAP shown for 18 patients (in one infant, no physiological data were recorded due to a technical error). Asterisks indicate significant differences compared with baseline (nCPAP 5 cm H_2_O) in single comparisons after correction for multiple testing. Overall, heart rate and Spo_2_/Fio_2_ ratio changed significantly over time. AU = arbitrary units; nCPAP = nasal CPAP; Spo_2_ = peripheral oxygen saturation.

**e-Figure 2:**
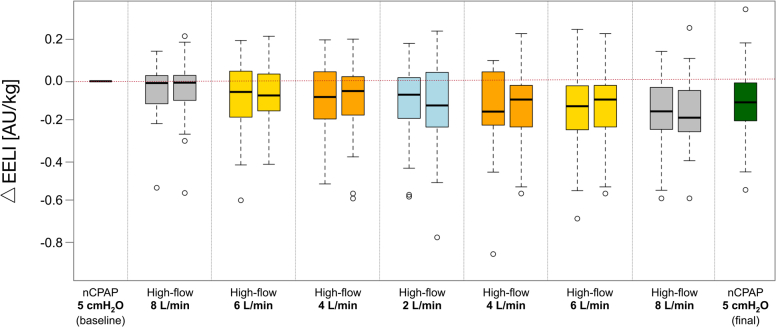


**e-Figure 3:**
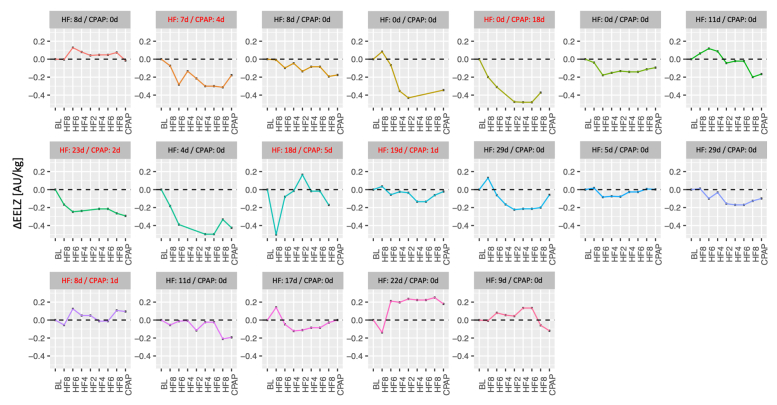


Changes in EELZ during the study are illustrated in Figure 2A and e-Figure 2. Compared with baseline, EELZ changed significantly throughout the study (Friedman test, *P* = .002). In post hoc tests, this change was mainly attributable to a loss of EELV when high flow was reduced to 6 and 4 L/min, when high flow was increased to 4, 6, and 8 L/min, and when infants were switched back to nCPAP (Table 2). The complete analysis for development of EELZ is provided in e-Table 2. ΔEELZ over time for individual patients is shown in e-Figure 3.

**Table 2:** Changes in EELZ, Heart Rate, Spo_2_/Fio_2_ Ratio, and Respiratory Rate Compared With nCPAP (Baseline) During the High-Flow Titration Sequence

Intervention	ΔEELZ, AU/kg	Adjusted *P* Value	ΔHeart Rate, beats/min	Adjusted *P* Value	ΔSpo_2_/Fio_2_	Adjusted *P* Value	ΔRespiratory Rate, min–1	Adjusted *P* Value
nCPAP (baseline)	Baseline	NA	Baseline	NA	Baseline	NA	Baseline	NA
High flow 8 L/min	−0.01 (−0.09 to 0.03)	.764	2 (−7 to 9)	1.000	−15 (−28 to −2)	.047	−12 (−18 to 0)	.012
High flow 6 L/min	−0.07 (−0.14 to 0.03)	.048	2 (−1 to 5)	.305	−16 (−28 to −4)	.043	−8 (−17 to 2)	.016
High flow 4 L/min	−0.05 (−0.16 to 0.02)	.048	7 (−3 to 9)	.305	−21 (−33 to 1)	.162	1 (−7 to 4)	.984
High flow 2 L/min	−0.10 (−0.2 to 0.02)	.070	5 (−1 to 13)	.305	−34 (−53 to −11)	.002	1 (−8 to 6)	.953
High flow 4 L/min	−0.09 (−0.2 to −0.02)	.004	2 (−3 to 11)	.552	−34 (−39 to 1)	.026	−4 (−15 to 8)	.312
High flow 6 L/min	−0.10 (−0.2 to −0.001)	.048	5 (−2 to 13)	.305	−19 (−39 to −3)	.043	−4 (−13 to 12)	.515
High flow 8 L/min	−0.20 (−0.2 to −0.04)	.021	10 (2 to 20)	.027	−27 (−44 to −9)	.039	−6 (−14 to 2)	.196
nCPAP (final)	−0.1 (−0.2 to −0.008)	.034	9 (1 to 13)	.105	−7 (−27 to 4)	.573	1 (−4 to 9)	.441

Values are depicted as median and interquartile range or as otherwise indicated. *P* values are adjusted for multiple testing and corrected using the Bonferroni-Holm method. AU = arbitrary units; EELZ, end-expiratory lung impedance; NA = not applicable; nCPAP = nasal CPAP; Spo_2_ = peripheral oxygen saturation.

### Changes in Other EIT Parameters

Except for a significant reduction in minute ventilation observed throughout the study (Friedman test, *P* = .004), the transition to high flow and the adjustments made to flow levels did not correlate with significant changes in EIT ventilation parameters (e-Table 3).

### Changes in Physiological Parameters

Changes in heart rate and Spo_2_/Fio_2_ ratio are shown in Figures 2B and 2C. There was a significant increase in heart rate over the duration of the measurement (Friedman test, *P* = .02), and only the comparison between baseline and the period when high flow was increased to 8 L/min reached statistical significance in post hoc analysis (Table 2). Spo_2_/Fio_2_ ratio changed significantly over time (Friedman test, *P* < .001) (Fig 2C) due to decreased ratios at high flow 8, high flow 6, high flow 2, high flow 4, high flow 6, and high flow 8 L/min compared with baseline (Table 2). Significant changes were also observed in respiratory rate (Friedman test, *P* = .009), Spo_2_ (Friedman test, *P* = .008), and Fio_2_ (Friedman test, *P* = .005) over the course of the study (e-Tables 3, 4; Table 2).

### Safety Outcomes

There were no adverse events during the study period, and no infants met the criteria for high-flow treatment failure.

## Discussion

The primary objective of this study in preterm infants was to describe changes in lung volume during the transition from nCPAP to high-flow therapy and at varying flow levels. Our findings indicate that high-flow weaning is associated with a gradual loss of lung volume, and only partial recovery is achievable with a stepwise escalation to preweaning high-flow levels. The transition back to nCPAP increases lung volumes; however, they remain below preintervention levels throughout the measured period. These changes in lung volume are accompanied by physiological responses, as indicated by changes in heart rate and Spo_2_/Fio_2_ ratio.

In this study in stable very preterm infants undergoing weaning from nCPAP to high flow, we observed a gradual loss of EELV throughout the intervention. We speculate that, for most of our study, distending pressures lower than the initial nCPAP were applied, potentially contributing to lung derecruitment even at higher flow rates. Indeed, flow rates varying between 2 and 5 L/min have been shown to generate pressures that consistently stay ≤ 5 cm H_2_O.^11^^,^^15^ In our study, we adhered to unit standards in high-flow treatment and did not actively close the infants’ mouth during data collection to enhance the generalizability of our findings, a factor that might have contributed to additional lung volume loss. Moreover, data on high-flow pressure generation in preterm infants come from in vitro models, animal studies, and small observational and crossover studies, which have used esophageal^21^^,^^22^ or nasopharyngeal^11^^,^^15^ pressures as a proxy for intrapulmonary pressures. However, changes in pulmonary physiology remain undetected when relying on pressure measurements at the nasopharyngeal or esophageal level or when using conventional tools for monitoring the behavior of the neonatal lung. EIT on the other hand quantifies impedance changes within a transverse chest slice, and thus, may reflect global and regional changes in lung volume more accurately.^13^ Also, compared with older infants or children, preterm infants exhibit low lung compliance and high chest wall compliance. They also have reduced collateral ventilation, with fewer interalveolar and interbronchiolar pores than older children.^23^ These differences render preterm infants more susceptible to intrapulmonary pressure fluctuations, which increases their risk of developing microatelectasis. This contributes to the gradual loss of EELV observed in our study, even with the application of higher flow rates. We speculate that initiating the weaning process with flow levels higher than the nCPAP level may enhance the likelihood of a successful transition off nCPAP. However, this needs validation through adequately powered clinical trials before implementation into clinical practice, particularly regarding the highly fluctuating applied pressures during high flow and potentially deleterious effects in form of air leaks.

We initially hypothesized that re-escalation of high-flow levels would be associated with a concurrent increase in lung volume. However, our observations revealed that during high-flow escalation, all EELV remained below those observed during high-flow weaning at identical flow rates. These changes in lung volume coincided with significant variations in heart rate and oxygenation, suggesting an altered physiological state of the lung between nCPAP and high flow, and during high flow at equivalent flow levels. We speculate that higher distending pressures than those provided by high flow would be necessary to maintain alveolar stability and to reopen collapsed lung tissue. This hypothesis would align with the physiological principles of the pressure-volume curve, indicating that larger pressures are needed to reopen atelectasis than to maintain already recruited lung volumes.^24^ Our findings may also provide a potential explanation for the findings of two large randomized controlled trials comparing high-flow therapy with nCPAP as primary respiratory support for preterm infants or as postextubation respiratory support.^5^^,^^6^ In such highly adaptive situations associated with lung volume inhomogeneity, both trials reported a higher rate of treatment failure in the high-flow group.

In addition to the observed loss in lung volume, we also noted a significant variability in lung volumes during the weaning and reescalation of high-flow levels in our stable preterm infants (e-Fig 3). This is consistent with the findings of 2 prospective studies using EIT in young children with bronchiolitis, demonstrating highly heterogeneous lung volume responses to flow rates ranging from 0.5 to 2 L/kg/min.^25^^,^^26^ Although some of the included children showed a reduction in lung volume despite the application of higher flow rates, a significant proportion displayed no relevant changes in lung volume. Also, our study targeted very preterm and very low birth weight infants and applied flow rates of up to 8 L/min. Both high flow rates and low infant weight are important determinants of the pressures generated by high flow and account for a significant amount of its variance.^15^

Consistent with the changes in lung volume, we also observed significant physiological responses throughout the intervention, including an increase in heart rate and a decrease in Spo_2_/Fio_2_ ratio. Both parameters are closely linked to the maintenance of optimal lung volumes and a balance between ventilation and perfusion in the lungs. As suggested by the U-shaped relationship between lung volume and pulmonary vascular resistance, lung volume losses due to inadequate alveolar expansion may lead to increased vascular resistance.^27^^,^^28^ This imposes an elevated afterload on the right ventricle, prompting an acceleration in heart rate to sustain cardiac output. As demonstrated previously, there is a strong correlation between EELV and oxygenation, explaining the decrease in Spo_2_/Fio_2_ ratio.^29^ Although both factors would be expected to increase the work of breathing, we did not assess this aspect throughout the procedure. Interestingly, the infants did not develop tachypnea, nor did they show alterations in tidal volume, as a potential clinical marker for the development of respiratory failure. This aligns with EIT measurements in children with bronchiolitis, revealing no clear relationship between EELV, effort of breathing, and tidal volume.^25^ Finally, other EIT parameters including silent spaces and center of ventilation remained comparable during the study. This indicates that despite changes in global lung aeration, ventilation distribution was not altered during the two modes of respiratory support and during different flow rates. Thus, the loss in lung volume seems to happen homogeneously with no consistent effect favoring one hemithorax.

Our study has various limitations. First, this was an observational single-center study, and the results may not be generalizable to different units, ventilators, interfaces, or settings. Second, we investigated only a small sample of infants, limiting the clinical validity of the data and preventing conclusions on relevant clinical outcomes. However, even with only 19 infants, we had 302 separate EIT recordings and 8,438 breaths to evaluate, which enhances the value of the data set. Third, EIT measures relative changes; thus, we cannot draw conclusions regarding absolute volume changes or pulmonary pressure changes during the procedure. However, EIT measurements can accurately describe changes over time as was the case in our study. Also, EIT measurements have been shown to correlate well with tidal volumes in animal studies and are representative of the whole lung in ventilated preterm infants.^8^^,^^23^^,^^24^^,^^30^, ^31^, ^32^ Fourth, our study using EIT was not intended to establish protocols for weaning infants off nCPAP or to guide individualized approaches for trials off respiratory support. The potential advantages of continuous bedside EIT monitoring on clinically significant outcomes require investigation through well-powered, randomized controlled trials. Until findings from such trials are established, the practical utility of EIT lung monitoring in the neonatal intensive care unit remains theoretical. Fifth, incorporating washout periods or randomizing infants to different flow rate sequences might have yielded different results. Additionally, the duration of each high-flow level was limited to 30 minutes, and although extended observation periods might have influenced the outcomes, existing literature suggests that respiratory baselines are typically achieved within 1 to 5 minutes after any alterations.^33^ Sixth, we only investigated a specific high-flow titration sequence and a consistent prone positioning during the transition from nCPAP to high flow and during high-flow weaning. Although we do not anticipate a divergent behavior of the lung, we cannot exclude variations in findings with alternative flow rates or supine positioning. Finally, nCPAP was inconsistently administered using nasal masks in some infants and short binasal prongs in others. Studies suggest that nasal masks may have more leakage than short binasal prongs, potentially affecting the applied nCPAP and the resultant lung volume changes between nCPAP and high flow.^34^

## Interpretation

Weaning stable, very preterm infants from nCPAP to high flow, followed by a reduction to lower flow levels, was associated with a decrease in EELV. Despite a stepwise escalation to preweaning flow rates, high flow failed to enhance lung volumes. These alterations in lung aeration were accompanied by changes in heart rate and oxygenation, reflecting a physiological shift in the lungs. Only the transition back to nCPAP increased EELV, albeit to levels still lower than those observed prior to the intervention. We hypothesize that pressures exceeding those generated by high flow are necessary to maintain alveolar stability and to restore lung volume to its preweaning state.

## Funding/Support

V. L. B. was supported by the Olga-Mayenfisch Foundation. V. D. G. was supported by the European Society for Pediatric Research, the Heuberg-Foundation, and the SwissLife Foundation. C. M. R. was supported by the EMDO Foundation. J. T. was supported by a Filling the Gap Grant of the University of Zurich. The EIT monitor was provided free of charge for the duration of the study by SenTec AG.

## Financial/Nonfinancial Disclosures

None declared.

## Supplementary Data

e-Online Data
