# Supplementary material for: Lung Volume Changes in Stable Preterm Infants Weaned From Nasal CPAP to High Flow: A Prospective Cohort Study
Source: CHEST Pulm. 2024 Aug 14;2(4):100094. doi: 10.1016/j.chpulm.2024.100094 (PMC13418385; doi:10.1016/j.chpulm.2024.100094)
Supplement: e-Online Data [file mmc1.docx]

**Title**: **Lung volume changes in stable preterm infants weaned from nCPAP to high-flow:** **a prospective cohort study**

**Short title**: FunkFlow study

**Authors**: Vanessa L. Büchler; Vincent D. Gaertner, MD; Janine Thomann, MD; Dirk Bassler, MD; Christoph M. Rüegger, MD

**Affiliations**: From Newborn Research Zurich (V. L. Büchler, J. Thomann, D. Bassler, and C. M. Rüegger), Department of Neonatology, University Hospital and University of Zürich, Zürich, Switzerland.

From the Division of Neonatology (V. D. Gaertner), Dr von Hauner Children’s Hospital, Ludwig-Maximilians-University Munich, Munich, Germany.

**Supplemental Material**

**e-Tables**

**e-Table 1:** Respiratory parameters computed and measured using electrical impedance tomography (EIT) according to the 2016 Translational EIT Development Study Group Consensus.^1^

| **Variable** | **Definition** |
| --- | --- |
| End-expiratory lung impedance (EELZ) | EIT signal at the end of expiration, a surrogate for end-expiratory lung volume (EELV). |
| End-expiratory lung volume (EELV) | EELV describes the gas volume which can contribute to gas exchange between two breaths. |
| Center of ventilation (CoV) | A functional EIT measure used to quantify the distribution of ventilation in relation to the anteroposterior (CoV_VD_) or right-to-left (CoV_RL_) chest diameter and expressed as percentage. |
| Silent spaces (SS) | Image regions with very low values of normalized tidal variation implying low ventilation (e.g. pixels with values <10%). |
| Impedance change (ΔZ) | ΔZ is defined as the peak-to-trough change in EIT signal during inspiration (ΔZ=EILZ–EELZ), a surrogate for tidal volume (V_T_). |

**e-Table 2.** *P*-values of post-hoc analyses for changes in EELZ during the transition from nCPAP (baseline) to high-flow and at different flow levels.

|  | nCPAP  (baseline) | High-flow  8 L/min | High-flow  6 L/min | High-flow  4 L/min | High-flow  2 L/min | High-flow  4 L/min | High-flow  6 L/min | High-flow  8 L/min | nCPAP  (final) |
| --- | --- | --- | --- | --- | --- | --- | --- | --- | --- |
| nCPAP (baseline) | NA | - | - | - | - | - | - | - | - |
| High-flow 8 L/min | 0.764 | NA | - | - | - | - | - | - | - |
| High-flow 6 L/min | 0.048 | 0.764 | NA | - | - | - | - | - | - |
| High-flow 4 L/min | 0.048 | 0.764 | 0.974 | NA | - | - | - | - | - |
| High-flow 2 L/min | 0.070 | 0.613 | 0.764 | 0.802 | NA | - | - | - | - |
| High-flow 4 L/min | 0.004 | 0.416 | 0.764 | 0.802 | 0.974 | NA | - | - | - |
| High-flow 6 L/min | 0.048 | 0.416 | 0.764 | 0.802 | 0.974 | 1.000 | NA | - | - |
| High-flow 8 L/min | 0.021 | 0.257 | 0.516 | 0.729 | 0.802 | 0.802 | 0.764 | NA | - |
| nCPAP (final) | 0.034 | 0.426 | 0.764 | 0.852 | 1.000 | 0.974 | 0.986 | 0.764 | NA |

Results for paired Wilcoxon tests with Bonferroni-Holm correction. nCPAP, continuous positive airway pressure.

**e-Table 3.** Global changes in other EIT and physiological parameters compared with nCPAP at baseline.

|  | High-flow  8 L/min | High-flow  6 L/min | High-flow  4 L/min | High-flow  2 L/min | High-flow  4 L/min | High-flow  6 L/min | High-flow  8 L/min | nCPAP  (final) | *P*-value |
| --- | --- | --- | --- | --- | --- | --- | --- | --- | --- |
| Other EIT parameters | | | | | | | | | |
| ΔCoV_RL_ (%) | -1.0 (-3.1 to 1.4) | -0.7 (-3.7 to 1.7) | -1.9 (-5.8 to 1.7) | 1.0 (-4.6 to 2.2) | 0.5 (-4.1 to 2.6) | 0.3 (-2.9 to 1.6) | 0.9 (-3.2 to 5.1) | 1.4 (-1.2 to 4.1) | 0.55 |
| ΔCoV_VD_ (%) | -0.2 (-1.6 to 0.7) | -0.3(-1.4 to 0.04) | 0.2 (-1.8 to 1.3) | -0.6 (-1.2 to 0.8) | -0.8 (-1.7 to 1.4) | -0.2 (-2.7 to 0.5) | -0.7 (-1.6 to 0.1) | 0 (-1.7 to 0.7) | 0.56 |
| ΔSS_NGD_ (%) | 0.0 (-0.1 to 0.3) | 0.0 (0.0 to 1.0) | 0.0 (0.0 to 0.3) | 0.0 (0.0 to 1.4) | 0.0 (0.0 to 1.1) | 0.0 (0.0 to 0.0) | 0.0 (0.0 to 1.6) | 0.0 (0.0 to 0.0) | 0.66 |
| ΔSS_GD_ (%) | 0.0 (-2.2 to 1.1) | 0.5 (0.0 to 1.4) | 0.0 (-1.4 to 1.5) | 0.3 (-0.8 to 0.8) | 0.6 (-1.4 to 2.6) | 0.0 (-0.8 to 1.2) | -0.5 (-1.6 to 1.6) | -0.5 (-1.9 to 0.3) | 0.37 |
| ΔZ, AU/kg | 0.01 (-0.01 to 0.01) | 0.0 (-0.0 to 0.01) | 0.0 (-0.01 to 0.02) | 0.01 (-0.01 to 0.02) | 0.0 (-0.02 to 0.01) | 0.0 (-0.01 to 0.01) | 0.0 (-0.01 to 0.01) | 0.0 (-0.01 to 0.02) | 0.41 |
| ΔMV, AU/kg/min | -0.7 (-1.4 to 0.2) | 0.02 (-1.4 to 0.7) | -0.2 (-1 to 0.5) | -0.1 (- 1 to 0.5) | -0.7 (-1.6 to 0.02) | -0.1 (-1.3 to 0.6) | -0.5 (-1.3 to 0.6) | -0.1 (-0.5 to 1) | 0.004 |
| Physiological parameters | | | | | | | | | |
| ΔSpO_2_ (%) | 0.1 (-0.2 to 1.9) | -1.2 (-3.0 to 0.0) | -2.9 (-4.4 to 1.8) | -3.0 (-5.5 to -0.7) | -1.8 (-6.5 to 1.0) | -1.2 (-3.6 to 0.0) | 0.0 (-3.5 to 2.4) | -2.3 (-5.4 to 0.4) | 0.008 |
| ΔFiO_2_ | 0.7 (0.03 to 1.4) | 0.4 (0.03 to 0.9) | 0.3 (-0.08 to 0.7) | 0.3 (0.0 to 1.7) | 0.6 (-0.08 to 0.7) | 1.0 (0.05 to 1.2) | 0.5 (0.0 to 1.3) | 0.0 (-0.2 to 0.3) | 0.005 |
| ΔRespiratory rate  (min^-1^) | -12 (-18 to 0) | -8 (-17 to 2) | 1 (-7 to 4) | 1 (-8 to 6) | -4 (-15 to 8) | -4 (-13 to 12) | -6 (-14 to 2) | 1 (-4 to 9) | 0.009 |

Depicted as median and IQR, except where otherwise specified. Global differences in medians over time were assessed using a Friedman’s test. nCPAP, nasal continuous positive airway pressure; CoV_RL_, center of ventilation along the right to left plane, CoV_VD_: center of ventilation along the ventral to dorsal plane; SS_NGD_, non-gravity-dependent silent spaces; SS_GD_, gravity-dependent silent spaces; ΔZ, impedance change as a surrogate for tidal volume (V_T_); AU/kg, arbitrary units per kilogram body weight; MV, minute ventilation; SpO_2_, peripheral oxygen saturation; FiO_2_, fraction of inspired oxygen.

**e-Table 4.** Physiologic parameters at baseline and during the high-flow titration sequence

| Parameter | nCPAP  (baseline) | High-flow  8 L/min | High-flow  6 L/min | High-flow  4 L/min | High-flow  2 L/min | High-flow  4 L/min | High-flow  6 L/min | High-flow  8 L/min | nCPAP  (final) |
| --- | --- | --- | --- | --- | --- | --- | --- | --- | --- |
| Heart rate (bpm) | 152  (142 to 158) | 154  (150 to 157) | 154  (147 to 161) | 155  (150 to 163) | 157  (150 to 166) | 155  (148 to 161) | 160  (154 to 163) | 161  (156 to 171) | 160  (152 to 168) |
| FiO_2_ | 21.9  (21.2 to 22.6) | 22.6  (22.0 to 23.3) | 22.8  (21.9 to 23.1) | 22.1  (21.6 to 23.7) | 22.1  (21.6 to 24.3) | 22.1  (21.3 to 23.5) | 22.9  (22.0 to 23.3) | 22.7  (21.3 to 23.7) | 21.8  (20.9 to 22.4) |
| SpO_2_ (%) | 95.7  (91.5 to 98.0) | 94.8  (91.7 to 98.0) | 93.3  (90.4 to 95.8) | 93.0  (90.9 to 95.7) | 93.2  (89.4 to 93.9) | 93.2  (89.8 to 95.1) | 94.8  (90.7 to 97.0) | 95.2  (89.8 to 96.5) | 92.7  (90.4 to 95.8) |
| SpO_2_ / FiO_2_ | 435.6  (407.5 to 453.8) | 420.2  (403.2 to 435.0) | 400.9  (393.2 to 441.4) | 418.2  (390.0 to 437.3) | 428.2  (360.3 to 433.3) | 422.7  (368.6 to 426.9) | 413.2  (386.8 to 432.2) | 420.8  (376.9 to 452.3) | 430.3  (395.7 to 444.0) |
| Respiratory rate (min-1) | 60  (51 to 68) | 46  (40 to 61) | 53  (42 to 61) | 54  (50 to 68) | 58  (52 to 62) | 55  (44 to 70) | 50  (45 to 71) | 53  (44 to 65) | 61  (52 to 71) |

Depicted as median and interquartile range. BPM, beats per minute; SpO_2_, peripheral oxygen saturation; FiO_2_, fraction of inspired oxygen.

**Supplementary Figure Legends**

**e-Figure 1.** Study workflow

After a baseline period on nCPAP at 5 cm H_2_O, infants were switched to high-flow support starting at 8 L/min for 30 minutes. The support level was reduced by 2 L/min every 30 minutes to 2 L/min, then increased by 2 L/min every 30 minutes back to 8 L/min. After 30 more minutes, infants were switched back to nCPAP with a PEEP of 5 cm H_2_O. During the intervention, EIT and physiological parameters (SpO_2_, FiO_2_, heart rate and respiratory rate) were measured continuously. Only data from 30-second intervals (boxes in darker blue and green color) were used for offline analysis. nCPAP, nasal continuous positive airway pressure; EIT, electrical impedance tomography; SpO_2_, peripheral oxygen saturation; FiO_2_, fraction of inspired oxygen; HR, heart rate; RR, respiratory rate.

**e-Figure 2.** Development of EELZ during the transition from nCPAP to high-flow at different flow levels.

Boxplots in the same color belong to 30-second intervals of artifact free tidal ventilation at the beginning and at the end of each high-flow level. ΔEELZ, change in end-expiratory lung impedance compared with nCPAP (baseline); nCPAP, nasal continuous positive airway pressure.

**e-Figure 3**. Development of ΔEELZ over time for individual patients.

ΔEELZ, change in end-expiratory lung impedance compared with nCPAP (baseline) for all individual patients. HF/nCPAP indicates the number of days on high-flow therapy and continuous positive airway pressure support after the intervention. Red font, infants in whom respiratory support had to be intensified following the intervention.

**Supplementary References**

1. Frerichs I, Amato M, Kaam Ah, et al. Chest electrical impedance tomography examination, data analysis, terminology, clinical use and recommendations: consensus statement of the TRanslational EIT developmeNt stuDy group. *Thorax* 2017;72(1):83.
